# Associations between *Mycobacterium tuberculosis* Strains and Phenotypes

**DOI:** 10.3201/eid1602.091032

**Published:** 2010-02

**Authors:** Timothy Brown, Vladyslav Nikolayevskyy, Preya Velji, Francis Drobniewski

**Affiliations:** United Kingdom Health Protection Agency, London UK (T. Brown, F. Drobniewski); Queen Mary College, University of London, London (V. Nikolayevskyy, P. Velji, F. Drobniewski); 1 These authors contributed equally to this article.

**Keywords:** Tuberculosis and other mycobacteria, microbial evolution, genetic polymorphisms, drug resistance, phenotypes, bacteria, research

## Abstract

To inform development of tuberculosis (TB) control strategies, we characterized a total of 2,261 *Mycobacterium tuberculosis* complex isolates by using multiple phenotypic and molecular markers, including polymorphisms in repetitive sequences (spoligotyping and variable-number tandem repeats [VNTRs]) and large sequence and single-nucleotide polymorphisms. The Beijing family was strongly associated with multidrug resistance (p = 0.0001), and VNTR allelic variants showed strong associations with spoligotyping families: >5 copies at exact tandem repeat (ETR) A, >2 at mycobacterial interspersed repetitive unit 24, and >3 at ETR-B associated with the East African–Indian and *M. bovis* strains*.* All *M. tuberculosis* isolates were differentiated into 4 major lineages, and a maximum parsimony tree was constructed suggesting a more complex phylogeny for *M. africanum*. These findings can be used as a model of pathogen global diversity.

## Abstract 2

This population-based study was used to investigate strong associations between phenotypes and genotypes.

Tuberculosis (TB), caused by bacteria of the *Mycobacterium tuberculosis* complex (MTBC), remains a global threat to human health, which causes an estimated 2 million deaths annually (*1*). No horizontal gene transfer has been reported in MTBC, and the genome is more highly conserved than other pathogenic bacteria (*2*). Nevertheless, genotyping tools have recently identified several polymorphisms in the MTBC genome that have provided insight into its evolution. Three major groups of MTBC genome alterations have been reported: single nucleotide polymorphisms (SNPs), large sequence polymorphisms (LSPs), and polymorphisms within repetitive sequences such as variable number tandem repeats (VNTRs). The first 2 groups mark irreversible genetic events and can be used to construct phylogenies for *M. tuberculosis* (*2*–*6*). An association between geographic region and *M. tuberculosis* families, defined by specific polymorphisms, has been demonstrated. This geographic structuring producing genetically, and perhaps phenotypically, distinct MTBC populations may contribute to differences in clinical features such as severity of disease or prevalence of extrapulmonary disease (*6*–*8*) and should be considered during the development of new drugs and vaccines.

Sreevatsan et al. divided MTBC strains into 3 principal genetic groups (PGG1–PGG3) based on SNPs in codon 463 of *katG* and codon 95 of *gyrA* (*2*). More recently, on the basis of polymorphisms in the *oxyR, katG,* and *rpoB* genes, strains have been divided into 5 lineages (I–IV and *M. bovis*); lineages I, III, and IV represent subgroups within PGG1, and lineage II corresponds to PGG 2 and 3 (*7*). By combining these markers with LSPs RD239, RD105, RD750, RD711, and RD702, a small 7bp deletion in the *pks15/1* gene and other SNPs, Gagneaux and Small were able to confirm these *M. tuberculosis* lineages and 2 lineages of *M. africanum* (*6*). The deletions RD9 and TbD1 are useful phylogenetic markers for other members of MTBC complex and ancestral *M. tuberculosis* strains (*3*). The loss and acquisition of repeats or spacers in the direct repeats region (*9*) does not appear to limit their value in biogeographic and phylogenetic studies (*10*,*11*).

Genotypic variation of MTBC strains at various geographic settings and significant associations between certain allelic variants at VNTR loci, MTBC lineages, and spoligotyping families have been reported (*7*,*12*–*15*). However, most studies used single genotyping methods on small populations or convenience samples. Population-based studies have focused primarily on areas of low- to middle-TB incidence, and it is unclear whether the results are universally applicable (*16*–*18*). Larger population-based studies on geographically diverse populations are needed to establish the phylogenetic, epidemiologic, and clinical relevance of such associations.

London accounts for nearly half of all TB cases in the United Kingdom (≈3,300 cases in 2006; incidence rate 44.8/100,000). Because 75% of these TB patients were born abroad (*19*), (Health Protection Agency update; www.hpa.org.uk), and clinical signs of disease develop within 2 to 3 years of arrival, we believe that the multicultural and diverse community in London provides a unique setting for studying the global biodiversity of MTBC. We aimed to establish whether MTBC isolates circulating in the London population are a useful model of global diversity, to determine the phylogenetic relevance of polymorphisms in repetitive regions of the MTBC genome, especially for *M. africanum* and its position in TB evolution, and to investigate associations between lineage and phenotype.

## Materials and Methods

### Study Design and Bacterial Isolates

One isolate from each of the 2,261 MTBC culture-positive patients was included in this prospectively designed population study. These isolates were collected from patients in all 30 London National Health Service hospitals between April 1, 2005, and March 31, 2006. Demographic data, including gender, date of birth, and country of birth were assigned to world regions according to an existing United Nations classification (*20*).

### Identification

Cultures were identified by using standard phenotypic identification tests (*21*) and molecular methods (Genotype Mycobacterium CM, AS, and MTBC kits; Hain Lifescience GmbH, Nehren, Germany) and the INNO LiPA Rif TB assay (Innogenetics, Ghent, Belgium) performed as recommended by the manufacturer. DNA was extracted from cultures using chloroform extraction as described (*22*). Isoniazid, rifampin, ethambutol, streptomycin, pyrazinamide, and ciprofloxacin susceptibilities were determined by using the resistance ratio method (*21*).

### Genotyping

All extracts were typed by using automated 15 mycobacterial interspersed repetitive unit–VNTR (MIRU-VNTR) fragment analysis (*23*–*26*). Clustered isolates were further genotyped by using an extended panel of 7 hypervariable VNTR loci (*27*). Data were exported to BioNumerics (Applied Maths, Sint-Martens-Latem, Belgium) for cluster analysis.

Spoligotyping was performed according to the manufacturer’s instructions (Isogen Lifescience, IJsselstein, the Netherlands) (*9*). Images were digitized and entered into BioNumerics software by using the BNIMA module (Applied Maths). Spoligotypes were assigned to families and subfamilies by using the online tools at http://cgi2.cs.rpi.edu/~bennek/SPOTCLUST.html (*10*). We have used the established spoligotyping families Beijing, Central Asian (CAS), East African–Indian (EAI), and *M. bovis* as lineage designations, as well as European American (EuroAm) (*13*,*28*) for the *M. tuberculosis* lineage, which includes the X, T, LAM, S, and Haarlem families.

### Other Methods

Detection of TbD1 and RD9 (*3*,*13*) was conducted by PCR fragment analysis (*3*). Reverse hybridization methods were used to analyze the 4 lineage-defining SNPs in 3 genes (*oxyR*^C37T^, *katG*^C87A^, *rpoB*^T2646G^, and *rpoB*^C3243T^) reported by Baker et al. (*7*) for selected isolates (n = 259) (*12*) and mutations in *katG*, *inhA*, and *rpoB* genes associated with drug resistance (*22*).

Data were analyzed by using Excel, BioNumerics (Applied Maths), SPSS 12.0 (SPSS Inc, Chicago, IL, USA) software and online interactive statistical tools (www.quantitativeskills.com/sisa). Categorical variables were analyzed by using relative risks (RRs), odds ratios (ORs), and the χ^2^ test. Discrimination power of genotyping methods was assessed using the Hunter-Gaston index (*29*).

## Results

### Diversity within the Study Population

We studied 2,261 isolates, representing 95.7% of all the bacteriologically confirmed TB cases reported in London from April 1, 2005, through March 31, 2006. Using routine phenotypic and genotypic methods, we identified 99.1% (2,241) as MTBC; the remaining 20 were too heavily contaminated for analysis.

Spoligotypes were generated for 98.8% (2,233) of the isolates; 656 types were identified, of which 458 were unique and 198 were shared by groups of 2–221 isolates. Isolates were assigned to families and subfamilies on the basis of their spoligotype by using the online tools at http://cgi2.cs.rpi.edu/~bennek/SPOTCLUST.html. All but 4 spoligotypes were assigned to >1 of 36 groups; 88.4% of isolates were assigned to a single spoligotyping family or subfamily. The remaining 11.6% were assigned to 2 families, albeit with given probabilities of <0.9. All the main spoligofamilies seen globally were represented within this population (Table 1).

**Table 1:** Analysis of associations between *Mycobacterium tuberculosis* phylogenetic lineages defined by SNP analysis and spoligotyping families in the group of isolates not classified using VNTR codes, UK*

Spoligotypes	Lineages (*6*,*7*) and relevant MIRU codes (*12*)				
	*M. tuberculosis*				*M. bovis*; 10–2, 40–2, C–5
	I/East Asian; 39–3, A–4, C–4	II/European American; 16–1,2,3, 39–2, B–1,2	III/EAI; 23–5, C–2	IV/Indo-Oceanic; 24–2, 26–2	
H37Rv, n = 2	0	2	0	0	0
Beijing, n = 13	**13**	0	0	0	0
LAM, n = 17	0	**17**	0	0	0
T, n = 53	1 600740007764671†	**51**	0	1 777200007403371†	0
Haarlem, n = 21	0	**20**	0	1 777777774000731†	0
EAI, n = 61	2 777777770003331† 477777377413771†	1 777734000000031†	0	**58**	0
CAS, n = 18	0	0	**18**	0	0
X, n = 9	0	**9**	0	0	0
S, n = 2	0	**2**	0	0	0
Family 33, n = 4	1	1	0	**2**	0
Family 35, n = 7	**7**	0	0	0	0
Family 36, n = 5	0	**5**	0	0	0
*M. bovis* BCG, n = 4	0	0	0	0	**4**
*M. africanum*, n = 35	**34**	1 710044706302261*	0	0	0

*SNP, single nucleotide polymorphism; VNTR, variable number tandem repeat; MIRU, mycobacterial interspersed repetitive unit; EAI, East African–Indian; LAM Latin American; CAS, Central Asian; BCG, bacillus Calmette-Guérin. European American includes the X, T, LAM, S, and Haarlem families. †Octal codes indicate spoligotyping patterns for isolates with disagreements between SNP- and spoligotype-defined lineages. Dominant families within each lineage are in **boldface**.

Isolates were cultured from a variety of body sites; 57% were of pulmonary origin. Where known, 60% of isolates were cultured from male patients and 40% from female patients; median age was 33 years. The COB was available for 1,381 (61.0%) patients; 1,157 (83.8%) were born in 89 countries outside the United Kingdom (Appendix Table). The population included representatives from all regions of the world (*20*).

### VNTR Data as Phylogenic Markers

**Figure 1:**
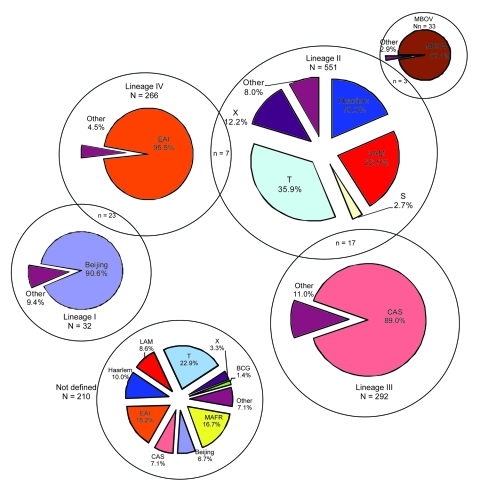
*Mycobacterium tuberculosis* complex lineages as determined by Gagneux et al. (*6*) and Baker et al. (*7*) defined by mycobacterial interspersed repetitive unit codes. MBOV, *M. bovis*; LAM, Latin American; CAS, Central Asian; EAI, East African–Indian; BCG, bacillus Calmette-Guérin; MAFR, *M. africanum*. The X, T, LAM, S, and Haarlem families are European American types.

The 22 MIRU-VNTR genotypes, generated for 2,261 isolates, resulted in 1,434 VNTR types representing the minimum number of independent strains within this population. Each type was designated an MTBC lineage on the basis of the VNTR types (*12*) (Figure 1). Where these lineages were ambiguous (n = 49), discordant to those suggested by spoligotype (n = 58), or not defined (n = 210), SNP analysis was performed to resolve these differences (n = 317). In all cases, the SNP analysis resolved the ambiguous VNTR lineage calling as 1 of the alternatives producing the ambiguity. The SNP-defined lineage of strains discordant between the spoligotype and VNTR agreed with the VNTR call in 74.0% of cases. Finally, among the strains for which the VNTR was unable to define any lineage, there was 94.0% agreement between the SNP and spoligotype-defined lineage (Table 1). All strains identified as *M. africanum* were placed in the nondefined group and had the SNP-1 genotype.

Spoligotyping gave a lineage that was confirmed by an independent marker (VNTR or SNP) in 96.3% of isolates. VNTR gave an unambiguous lineage in 77.9% of strains; of these, 99% were confirmed by an independent marker (spoligo or SNP). Allelic variants were sought at each VNTR locus that best described each spoligofamily; those giving the highest sensitivities and specificities are shown in Table 2. The highest sensitivities were seen in the LAM 1, LAM 10, and Beijing families, which suggests their highly clonal and homogeneous nature. Several allelic variants showed strong associations with spoligo families, with >5 copies at ETR-A, >2 copies at MIRU24, and >3 copies at ETR-B associated with EAI and *M. bovis* (RR 2.99, 95% confidence interval [CI] 2.51–3.56; RR 6.29, 95% CI 4.87–8.12; and RR 3.21, 95% CI 2.63–3.93, respectively), >3 copies at MIRU4 and 2 copies at MIRU26 with EAI (RR 2.31, 95% CI 1.98–2.70; and RR 12.8, 95% CI 8.41–17.90, respectively), and 4 copies at MIRU23 with *M. africanum* and *M. bovis* (RR 220.3, 95% CI 82.07–591.50).

**Table 2:** Associations between *Mycobacterium tuberculosis* MIRU15 profiles and spoligotyping families and subfamilies, UK*

Spoligotype families	MIRU15 allelic variants															Se, %	Sp, %
	2	4	10	16	20	23	24	26	27	31	39	40	A	B	C		
Beijing	2	2	2; 3	2–4	2	5; 6	1	5–8	1–3	5	2–4	1–4	3; 4	2	4	80.0	99.9
CAS	2	2	Any	3-5	2	5	1	Any	3	4; 5	2; 3	1–4	3; 4	2	2	72.6	98.3
EAI1	2	Any	2-6	1–4	2	5	1	4–6	3	3; 5	2; 3	<5	2–4	2	2–4	77.0	85.4
EAI2	2	>3	4; 5	2; 3	2	6	2	2–4	3; 4	3–5	3; 4	2; 3	4; 6	>2	4	73.3	99.9
EAI3	2	>2	3; 4	1–5	2	6–8	2	2	3	4–6	2; 3	Any	>4	1	3; 4	77.8	99.9
EAI4	2	2–6	4	2; 3	2	5; 6	2	2	1; 3	Any	1; 3	2; 3	>6	2–4	4	58.8	98.8
EAI5	2	1–9	3–6	2–4	2	>3	1–3	2	1–3	2–7	1–3	1–4	>4	2–7	2–4	75.0	98.3
MAF	2	2; 3	4–7	Any	2	4	1; 2	3–5	2–4	Any	2	1; 2	>3	2–4	4; 5	63.4	99.9
MBOV	2	1–3	2	3	2	4	2	5	2; 3	3	2	2	5	5–7	5	72.1	99.9
Haarlem1	2	2	2–6	2–4	1; 2	3; 5	1	4–7	3	3	2	2–5	2; 3	1; 2	3–5	66.7	89.2
Haarlem2	2	2	4; 5	1–3	1; 2	3–6	1	4; 5	1–3	3	2	1–4	2; 3	1; 2	3; 4	71.0	91.5
Haarlem3	2	2	2–6	1–4	1; 2	3; 5	1	4–6	3	2; 3	2	1–4	2–4	1; 2	2; 3	52.9	94.6
LAM1	2	2	3; 4	2	2	6	1	5; 6	2; 3	3	2	1	2	2	4	80.6	99.9
LAM10	2	2	2–4	2–4	1; 2	5	1	3–5	3	3	2	1–4	2–4	2	4	87.4	92.4
LAM3	2	2	4	2; 3	2	5; 6	1	4; 5	3	3	2	3	1; 2	2	2; 4	78.4	99.9
LAM5†	2	2	4	3	2	5	1	9	2	4	2	4	2	2	4	100.0	100.0
LAM7†	2	2	4	3	2	6	1	3	3	3	2	3	2	2	4	50.0	100.0
LAM8	2	2	2–5	1; 3	2	5 6	1	4; 5	1–3	3	2	1	2	1	4	66.2	99.4
LAM9	1; 2	2	2–4	1–3	1; 2	5–8	1	4–6	2; 3	2–4	2	Any	1–4	1; 2	2–6	72.7	77.1
S	2; 3	2; 3	3	2; 3	1–3	5; 6	1	4–6	3	2; 3	2	2; 4	4	2	4	44.4	99.8
T1	2	2; 3	Any	Any	2	5; 6	1	<7	2; 3	2–4	2	Any	2–4	2	2–5	54.5	85.8
T2†	2	2	3; 5	3	2	5; 6	1	5	3	2; 3	2	1; 3	2; 3	2	3–5	100.0	98.3
T3	2	2	3–5	1–3	1; 2	5; 6	1	1; 5	3	3	2	2–5	3	2	4	60.5	95.7
T4	1; 2	2–4	2–4	3	1; 2	5; 6	0–2	4–5	2; 3	2–4	2	2–4	2; 4	1; 2	2–5	46.2	88.3
X1	2	2	3–6	3	1; 2	5; 6	1	1–7	3	2–4	2	2–7	3; 4	1; 2	2–5	61.4	92.3
X2	1; 2	1; 2	4	3	2	5	1	4–8	3; 4	2; 3	2	1–4	2; 3	2	2; 4	79.5	99.3
X3	2	2	3; 4	2; 3	2	5	1	4; 5	3	2; 3	2	2–5	3	2	3	66.7	99.0

*MIRU, mycobacterial interspersed repetitive unit; Se, sensitivity; Sp, specificity; CAS, Central Asian; EAI, East African–Indian; MAF, *M. africanum*; MBOV, *M. bovis*; LAM, Latin American. The X, T, LAM, S, and Haarlem families are European American types. Any means any family or subfamily. Only strains with no secondary assignations to spoligotype groups were used for calculating associations. †Due to a small number of isolates in these families, Se and Sp values are calculated for illustrative purposes only.

The presence of 2 copies at MIRU24 appears to be a good marker for EAI *M. tuberculosis* and non*–M. tuberculosis* members of the MTBC. This marker (number of copies in the locus MIRU24) was investigated in this population by using the occurrence of the deletions RD9 and TbD1, which have previously been used as markers to distinguish these groups (Table 3). All 41 isolates identified as *M. africanum* by spoligotype were also analyzed in this manner, 11 of which contained a single copy of MIRU24; 296 *M. tuberculosis* isolates containing single and double copies of MIRU24 were analyzed as controls.

**Table 3:** Association between *Mycobacterium tuberculosis* spoligotypes, deletions, and allelic variants in the locus MIRU24, UK*

Deletion mapping and VNTR typing results	Spoligotype families			
	*Mycobacterium bovis*, n = 14	*M. africanum*, n = 41	*M. tuberculosis*	
			EAI, n = 241	Other, n = 55
TbD1+	14	41	239	19
TbD1–	0	0	2	36
RD9+	0	1	235	46
RD9–	14	40	6	9
MIRU24>2	14	30	240	24
MIRU24<1	0	11	1	31

*MIRU, mycobacterial interspersed repetitive unit; EAI, East African–Indian; VNTR, variable number tandem repeat.

**Figure 2:**
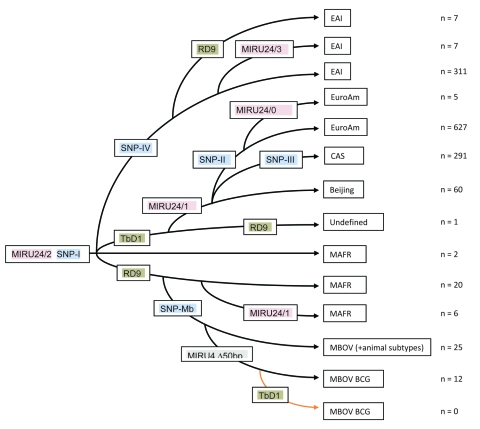
Maximum-parsimony tree constructed based on 3 independent sets of markers: large sequence polymorphisms (LSPs), single nucleotide polymorphisms (SNPs), and number of repeats in the locus 24 using the following assumptions: 1) SNPs are irreversible unique events; 2) LSPs are irreversible rare events; 3) spoligotypes are not produced by convergent events; and 4) variable number tandem repeat (VNTR) loci can both acquire and lose repeats. EAI, East African–Indian; MIRU, mycobacterial interspersed repetitive unit code; EuroAm, European American; CAS, Central Asian; MBOV, *M. bovis*; MAFR, *M. africanum*; BCG, bacillus Calmette-Guérin. The X, T, LAM, S, and Haarlem families are European American types.

The deletion TbD1 was present in all EuroAm, CAS, and Beijing strains examined as well as some other *M. tuberculosis* isolates and absent from all *M. africanum* isolates. The deletion RD9 was present in all *M. bovis* strains as well as some EAI and most *M. africanum* strains but absent from all other strains. Both deletions were absent from most EAI and some *M. africanum* strains (Table 3). Absence of RD9 deletion and 2 copies in MIRU24 was strongly associated with EAI spoligotype (RR 15.1, 95% CI 9.49–23.89). MTBC strains with the RD9 intact and 2 copies in MIRU24 included both *M. bovis* and *M. africanum* spoligotypes, whereas strains with the RD9 intact and 1 copy in MIRU24 formed a specific group of *M. africanum* originating presumably from the Indian subcontinent. Using this data, and the SNP 1-MB and the MIRU24 enumeration data, we constructed a maximum-parsimony tree as shown in Figure 2.

### Associations between Phylogenic Groups and Phenotype

Strong associations were seen between patient’s country of origin and the spoligo family of the isolate (Appendix Table): CAS and EAI families dominated in patients born on ISC (RR 2.4, 95% CI 2.02–2.74) as did Beijing and EAI families in patients born in Southeast Asia (RR 4.8, 95% CI 2.70–8.54). EAI families were seen in 80.4% of isolates from patients born in East Africa and the ISC. The *M. africanum* family dominated in patients born in West Africa (RR 3.67, 95% CI 1.52–6.50). In contrast, LAM and Haarlem isolates were infrequently seen in patients born on the ISC (4.5% and 5.4%) and Southeast Asia (3.4% and 6.9%). T family isolates, one of the genetic groups determined by spoligotyping, were distributed evenly across all regions except Southeast Asia, where they were infrequently seen. No association between lineage or spoligo family and pulmonary versus extrapulmonary site was seen in the present study.

Susceptibility to rifampin, isoniazid, ethambutol, streptomycin, and pyrazinamide was evident for 98.9% (2,236) of the isolates. Of these isolates, 84.3% were sensitive to all, 8.2% were isoniazid resistant, 5.4% streptomycin resistant, 1.5% rifampin resistant, 0.7% ethambutol resistant, 0.5% pyrazinamide resistant, and 1.2% multidrug resistant.

Associations between spoligotype families and drug resistance of MTBC strains were analyzed by determining the minimum number of independent clones and the minimum number of resistance acquisition events within this population. VNTR15 cluster analysis was performed on all isolates (n = 2,261) to identify a single representative of each unique genotype. This analysis resulted in 1,166 unique types.

When isolates shared a genotype but differed in susceptibility to a given drug, resistant and sensitive isolates were analyzed because the resistant isolate must have undergone a genetic event and acquired a unique genotype. When genotypes for loci associated with isoniazid and rifampin resistance had been determined and >1 type was present in a cluster, 1 of each type was included. Where members of a cluster and its nearest neighbor were resistant, this was considered as a single acquisition event and only a single member was included. The resulting numbers divided between spoligotype families are shown in Table 4.

**Table 4:** Minimum number of unique types seen within each *Mycobacterium tuberculosis* spoligotype family, by resistance or susceptibility to 5 antimicrobial drugs, United Kingdom*

Spoligotype family	No. types, by drug resistance or drug susceptibility											
	STR-R	STR-S	INH-R	INH-S	ETH-R	ETH-S	RIF-R	RIF-S	PZA-R	PZA-S	MDR+	MDR–
Beijing	10	40	13	38	3	43	5	43	1	45	5	45
CAS	14	202	30	198	1	206	3	206	2	206	3	206
EAI	8	244	23	234	3	247	4	247	1	248	4	247
European American	45	451	59	441	5	475	19	467	6	474	10	470
*M. bovis* BCG	0	6	2	4	1	5	1	5	2	3	1	5
Family 33–36	5	39	4	41	1	43	1	43	0	44	1	44
*M. africanum*	2	22	0	22	0	22	0	22	0	22	0	22

*STR, streptomycin; R, resistant; S, susceptible; INH, isoniazid; ETH, erythromycin; RIF, rifampin; PZA, pyrazinamide; MDR, multidrug-resistant; CAS, Central Asian; EAI, East African–Indian.

The *M. bovis* BCG family was associated with pyrazinamide (p<0.0001) and ethambutol resistance (p = 0.0009). Beijing family strains were associated with multidrug resistance (p = 0.0001), isoniazid (p = 0.0019), and rifampin (p = 0.0027) resistance. Associations were seen between streptomycin resistance and the Beijing family (p = 0.0008) and between pyrazinamide (p = 0.0079) and streptomycin (p = 0.008) resistance and the LAM1 family.

## Discussion

Several approaches have been used to study the global diversity of MTBC. One approach is to construct a global sample of isolates from reference collections around the world (*19*,*30**,**31*). In this instance, the degree of confidence as to geographic origin of an isolate is high, but bias occurs 1) where variety is limited to sites with which investigators have contact and 2) sites with high TB transmission, which often lack adequate facilities for bacteriologic culture. A second approach is to study isolates derived from a population at a single geographic location whose members have diverse geographic origins throughout the world (*19*,*30**,**31*). In this instance, where country of birth data are used to indicate the geographic origin of an isolate, the degree of confidence in this data may be lower, but MTBC isolates can be sampled at a wider range of geographic locations particularly from high TB incidence areas that have poor bacteriologic isolation facilities. Furthermore, additional data such as antimicrobial drug susceptibility and site of infection, useful for association studies, are retained and the quality of the data is ensured.

London is a cosmopolitan city where up to 30% of the population is foreign born (www.neighbourhood.statistics.gov.uk), among whom 75% of TB cases are seen (*19*; HPA update, www.hpa.org.uk); a similar situation has been reported in New York and Paris (*19*,*30**,**31*), although London TB notification rates (44.8 cases/100,000 population in 2006) are generally higher than those for other high-income cities. We believe that London provides a suitable setting for studying global MTBC diversity because our study shows that TB patients came from 89 different countries of origin, representing all regions of the world (*20*), including areas that the World Health Organization has defined as having a high incidence of TB. The bacterial diversity within this population is shown by the presence of all the main spoligofamilies, although not all lineages are equally represented. Our study shows a disproportionate representation of patients from different regions; relatively small numbers were from the Americas.

Recent advances in molecular genotyping and comparative genomics have demonstrated that the level of genetic variation in the MTBC may have been substantially underestimated. Rapidly evolving genomic regions such as VNTR and the direct repeat region have been exploited for epidemiologic studies, whereas irreversible events recorded by SNPs and LSPs are of phylogenetic value (*3*,*5*–*7*). Associations between polymorphisms in rapidly evolving genomic regions (VNTR or direct repeat region) and the SNP and LSP markers have been described (*6**,**12**,**13**,**28**,**32**–**34*). If the nature of these relationships could be clearly defined, large studies could be performed by investigating databases containing routine VNTR data.

Where lineages indicated by SNP and LSP analysis are congruent with spoligotype family names, we have retained these (as for CAS, EAI, Beijing, *M. africanum* and *M. bovis*); for the lineages containing LAM, Haarlem, X,T, and S spoligo families, we have used the lineage designation EuroAm as suggested elsewhere (*6*). We have previously reported 10 VNTR loci (ETR A,B,C; MIRU10,16,23, 24, 26,39,40) (*12*) capable of differentiating the MTBC into 4 lineages (I–IV) and *M. bovis* (*7*).

VNTR analysis showed that 1,174 (81.9%) of 1,434 independent strains could be grouped unambiguously into 5 lineages. When the remainder were grouped by using the SNP analysis, a good correlation was seen between lineage and spoligotype family or group of families (Figures 1, 2).

Discrepancies between lineage and spoligo family mainly resulted from limitations imposed by the family designation software, choice of genetic targets analyzed, or overlapping rules defining some spoligo families. Strains belonging to families 33–36 and EAI 1 appeared in multiple lineages. These spoligotype families were designated as low probability, which suggests that the model spoligotype was detecting unrelated events in different families. In rare cases, discrepancies will be seen where genetic events converge to give identical types in unrelated strains. In the present study this can be seen when multiple lineages are indicated by VNTR or spoligotypes.

Discrepancies will also occur where the VNTR/SNP system fails to distinguish between spoligotype families. The most striking of these are the strains identified as *M. africanum* by spoligotype but as the Beijing lineage because of the presence of SNP1. We resolved this problem by constructing a maximum-parsimony tree (Figure 2) using the 5 SNP, LSP, and MIRU24 repeat numbers. The MIRU24 repeat numbers appear to play a phylogenetic role, as shown in this study (Table 3) and previous studies (*13*,*15*) in which >2 repeats are markers for EAI2–EAI5 (but not EAI1), *M. tuberculosis,* and *M. bovis* strains. In its construction, we made the assumptions that SNPs mark irreversible unique events and that VNTR loci can acquire and lose repeats. A BCG strain isolated from a patient from London (not included in this study) contained the TbD1 deletion, demonstrating clearly that these deletion events are not unique. Therefore, the assumption that LSPs are infrequent irreversible events was made. The strains in this study are of human origin and therefore are mainly *M. tuberculosis* and *M. africanum*, hence the focus of the phylogenetic scenario. The tree shown here is concordant with previous scenarios (*3*,*6*) differing only in the diversity seen in strains identified as *M. africanum*. All these strains contained SNP1 and were identified on the basis of the loss of spoliogotype spacers 8, 9, and 39 but contained either 1 or 2 copies of MIRU24 and the presence and absence of RD9, resulting in 3 types. The absence of the TBD1 deletion distinguishes *M. africanum* strains from Beijing strains.

EAI strains may represent the ancestral MTBC type (*6*,*15*). The data presented here suggest that *M. africanum* competes for this distinction. The types containing 2 copies of MIRU24, with and without RD9 originate exclusively from West Africa, suggest that these may be indigenous to this region. *M. africanum* species have traditionally been phenotypically subdivided into 2 subgroups, Type 1 (West African) and Type 2 (East African) (*34*). Recent genetic analysis suggests that *M. africanum* Type 2 (East African) is a phenotypic variant of *M. tuberculosis* and relatively distant from *M. africanum* Type 1 (West African), which is characterized by a deleted RD9, an intact TbD1 region, and specific SNPs in *katG* and *gyrA* genes (*35*,*36*). Our data suggest a more complex phylogeny of *M. africanum* Type 1 (West African). This phylogeny is complicated further by strains with a deleted RD9 and a single copy of MIRU24 originating predominantly from the Indian subcontinent.

The VNTR numbers seen within each spoligo family are shown in Table 3. From these data, lineage-dependent VNTR locus plasticity can be seen. This plasticity ranges from 7/15 loci showing variation within the CAS to 14/15 showing variation in the EuroAm lineage. VNTR loci such as MIRU10 and 16 show variation across all families, whereas MIRU27 shows variation in CAS alone. The distribution of repeat numbers at each locus within each lineage suggests the variation seen has arisen by stepwise mutations of a lineage founder strain. It is likely that the VNTR profiles used to predict spoligotype family at the highest specificity (Table 2) represent this type.

Using country of birth as a surrogate for geographic origin of an infecting strain, we saw strong associations with the lineage/spoligo family of isolates (Appendix Table). The data here confirm published data that Beijing strains were associated with patients originating from Southeast Asia; EAI with patients from Southeast Asia, Indian subcontinent, and East Africa; CAS with patients from the Indian subcontinent; and EuroAm with a global distribution of patients (*7*,*32*). This global geographic structuring may explain the apparent geographic variation in efficacy of the *M. bovis* BCG vaccine.

It has been long questioned whether there is an association between site and progression of infection and bacterial genotype; some evidence supports this association (*37**,**38*). Our study showed no association between lineage or spoligo family and site of infection.

That the *M. bovis* family was associated with pyrazinamide resistance would be expected because resistance is a defining characteristic for most of the group (although not for *M. bovis* subsp. *caprae)*. Beijing family isolates were associated with multidrug resistance and streptomycin resistance. The association with multidrug resistance has been reported (*8*), but the evidence presented here is particularly compelling, given that all strains used in the analysis were individual types. The value of this approach was demonstrated by analyzing LAM10 isolates, a family to which a highly successful clone of isoniazid-resistant *M. tuberculosis* responsible for >250 cases in northern London (*38*) belongs. Eight isolates were identified in this study. When all isolates belonging to this group were analyzed, LAM10 was strongly associated with isoniazid resistance (p<0.00001), but when a single representative of each cluster was used this association disappeared. The Beijing lineage would appear to have a predisposition toward the acquisition of drug resistance rather than the drug-resistant clones being transmitted more frequently. The extent of the geographic regions used in the association study make it unlikely that this predisposition is entirely due to local TB control and treatment practices.

## Supplementary Material

Appendix Table Distribution of Mycobacterium tuberculosis spoligotyping families by country/region of origin (N = 2,261), United Kingdom*
