# Supplementary material for: Associations between Mycobacterium tuberculosis Strains and Phenotypes
Source: Emerg Infect Dis. 2010 Feb;16(2):272–80. doi: 10.3201/eid1602.091032 (PMC2958017; doi:10.3201/eid1602.091032)
Supplement: Appendix Table — Distribution of Mycobacterium tuberculosis spoligotyping families by country/region of origin (N = 2,261), United Kingdom* [file 09-1032_appT-s1.pdf]

Appendix Table. Distribution of *Mycobacterium tuberculosis* spoligotyping families by country/region of origin (N = 2,261), United Kingdom*

Country/region of origin	*M. africanum*, n = 41	*M. bovis* BCG, n = 43	Beijing, n = 130	CAS, n = 552	EAI, n = 357	H37Rv, n = 9	Haarlem, n = 205	LAM, n = 347	S, n = 18	T, n = 366	X, n = 104	Fam33–36, n = 65	Unknown/ not done, n = 24
UK, n = 224	2	1	19	32	13	2	29	**50**	1	40	21	7	7
Western, southern, and northern Europe, n = 31	–	–	1	1	–	–	**9**	**9**	–	7	1	2	1
Eastern Europe, n = 22	–	–	2	2	–	–	6	2	–	**8**	–	2	–
Eastern Africa, n = 344	–	1	13	**82**	67	2	18	59	4	67	14	14	3
Middle Africa, n = 30	1	–	–	–	–	–	4	7	–	**14**	3	1	–
Northern Africa, n = 15	–	1	–	–	–	–	**5**	4	1	2	1	1	–
Western Africa, n = 84	19	1	1	1	6	1	8	**32**	–	10	4	1	–
South Africa, n = 28	–	–	5	–	1	1	–	**10**	1	4	5	–	1
Western Asia, n = 15	–	1	–	1	1	–	5	1	1	5	–	–	–
South-central Asia, n = 34	–	–	1	13	1	1	11	1	–	4	1	1	–
Indian subcontinent, n = 463	3	–	19	**203**	122	–	25	21	3	48	6	10	3
Eastern and Southeast Asia, n = 58	–	–	**27**	1	21	–	4	2	–	2	–	1	–
Caribbean, n = 21	–	–	1	2	1	–	3	**6**	–	4	2	2	–
South America, n = 8	–	–	–	–	1	–	2	2	–	**3**	–	–	–
Other, n = 4	–	–	–	–	1	–	–	–	–	**3**	–	–	–
Not known, n = 880	16	38	41	214	122	2	76	141	7	145	46	23	9

*CAS, Central Asian; EAI, East African–Indian; EuroAm, European American; LAM, Latin American. EuroAm includes the X, T, LAM, S, and Haarlem families. –, no strains belonging to a given spoligotyping family found in patients born in a given region. Dominant types in each region are in **boldface.**
